# Supplementary material for: Green Synthesis of Phosphorescent Carbon Dots for Anticounterfeiting and Information Encryption
Source: Sensors (Basel). 2022 Apr 12;22(8):2944. doi: 10.3390/s22082944 (PMC9026503; doi:10.3390/s22082944)
Supplement: Supplementary file 1 [file sensors-22-02944-s001.zip › Supporting Information.pdf]

Supporting information

Green synthesis of phosphorescent carbon dots for anti-counterfeiting and information encryption

Mingming Cheng, [1] Lei Cao, [1] Hanzhou Guo, [2] Wen-Fei Dong, [1] and Li Li [1,*]

[1] School of Biomedical Engineering (Suzhou), Division of Life Sciences and Medicine, University of Science and Technology of China, Hefei, China. cmm13862591726@mail.ustc.edu.cn (M. C.); cl1221@mail.ustc.edu.cn (L. C.); wenfeidong@sibet.ac.cn (W. D); lil@sibet.ac.cn (L. L.)

2 Changchun Guoke Medical Engineer and Technology Development Co., Ltd., Changchun, China; guohanzhou1@163.com (H. G.)

* Correspondence: lil@sibet.ac.cn; Tel: +86-15995709126

Chemicals and materials

Thiamine hydrochloride (Vitamin B1), ethylenediamine (EDA), and boric acid (BA) were purchased from Titan Scientific (Shanghai, China). Ultrapure water was used throughout the whole experiment.

Instrumentation

High-resolution transmission electron microscopy (HR-TEM) pictures were collected at 100 kV using a TECNAI G2 microscope (FEI, USA). The image of scanning electron micrographs (SEM) was measured by XL-30ESEM FEG scanning electron microscope (FEI, USA). FTIR spectra were conducted with a VERTEX 70 FT-IR spectrometer (Bruker, Germany). The X-ray photoelectron spectroscopy (XPS) spectra were acquired using an ESCALAB 250Xi spectrometer (ThermoFisher, USA). A Rigaku Minister apparatus was used to generate the X-ray diffraction (XRD) patterns (Tokyo, Japan). The UV absorption spectra were performed via a Hitachi UV2450 spectrophotometer (Tokyo, Japan). The fluorescence spectra were obtained using an F97Pro FL spectrophotometer coupled with a 1.0 cm quartz cell (Lengguang Technology, Shanghai). Also, the fluorescent and phosphorescent lifetime were analyzed at room temperature using an FLS-1000 fluorescence spectrophotometer (Edinburgh, UK).

Experimental Section

Synthesis of VB1-CDs

In general, 0.5 g of vitamin B1 was dissolved in 10 ml of ultrapure water initially, and then 150 µl of EDA was dropped into the solution and ultrasonically dispersed well. Subsequently, the solution was transferred to a 50 ml poly(tetrafluoroethylene) lined autoclave and reacted at 180°C for 8 hours in a drying oven. After the reaction, the centrifuge (8000 rpm, 10 min) removes the pellets and prepares them for usage.

Synthesis of PCDs

Basically, aspirate 50 µl, 100 µl, 500 µl, 1000 µl of VB1-CDs solution into a small beaker, add 20 ml of ultrapure water, stir evenly, then add 3 g of boric acid, cover with tin foil, and place in a drying oven to react at 180°C for 5 hours, grinding the material into a powder.

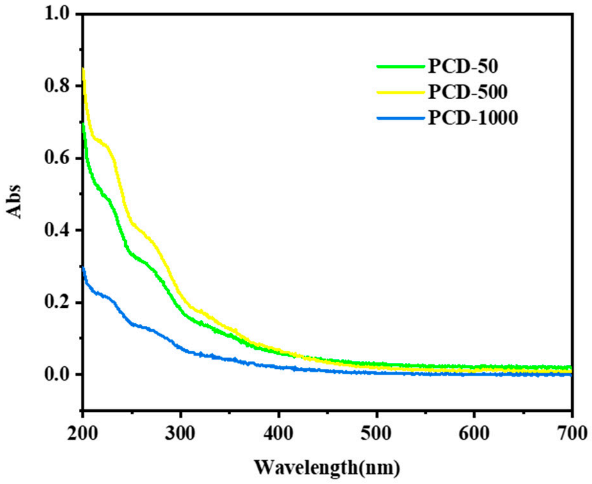

Figure S1 UV–Vis absorption of PCD50, PCD500 and PCD1000, respectively.

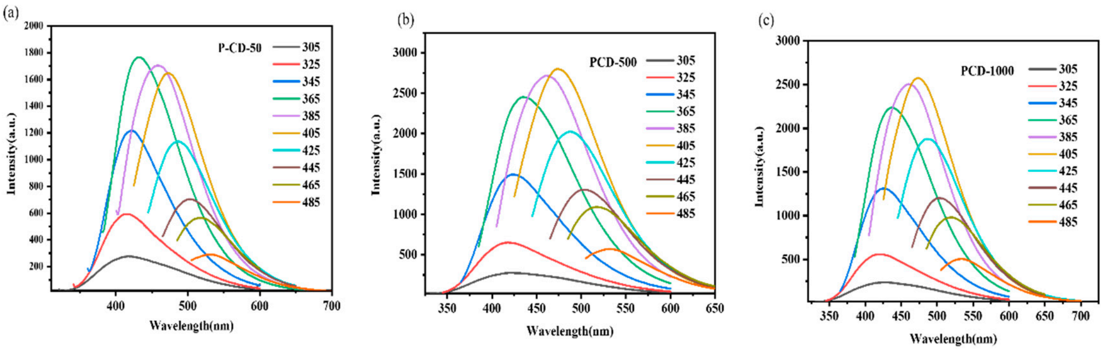

Figure S2 PL emission spectra of (a) PCD50, (b) PCD500 and (c) PCD1000 aqueous solution, respectively.

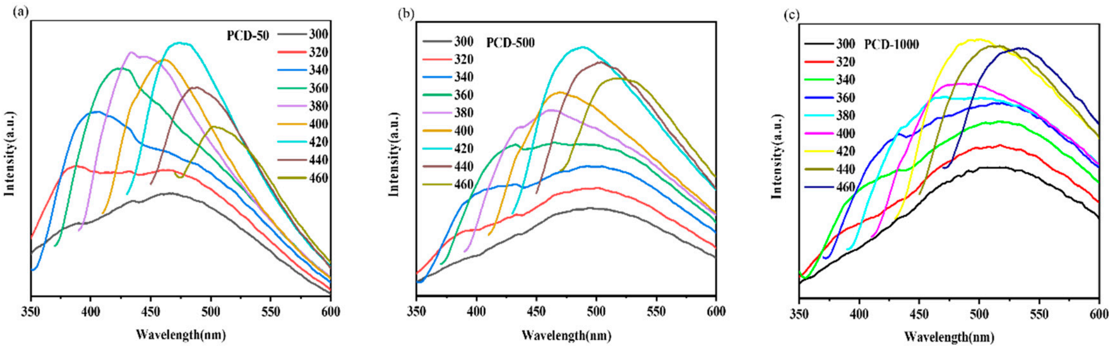

Figure S3 PL emission spectra of (a) PCD50, (b) PCD500 and (c) PCD1000 solid, respectively.

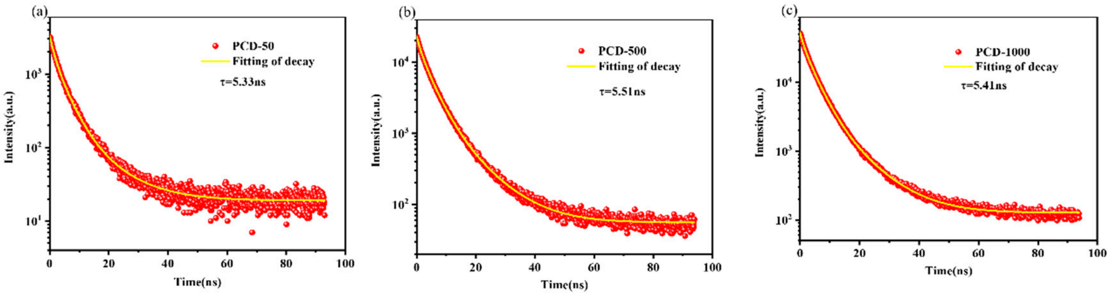

Figure S4 Fluorescence lifetime decay of (a) PCD50, (b) PCD500 and (c) PCD1000 aqueous solution respectively.

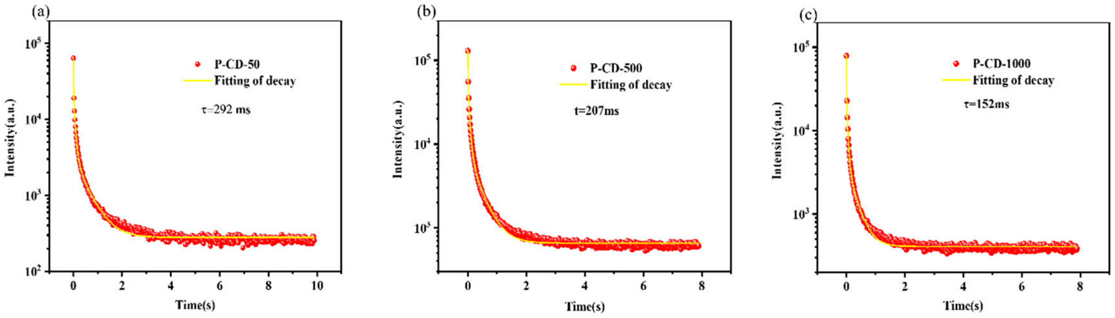

Figure S5 RTP lifetime decay of (a) PCD50, (b) PCD500 and (c) PCD1000 under the excitation of 365 nm, respectively.

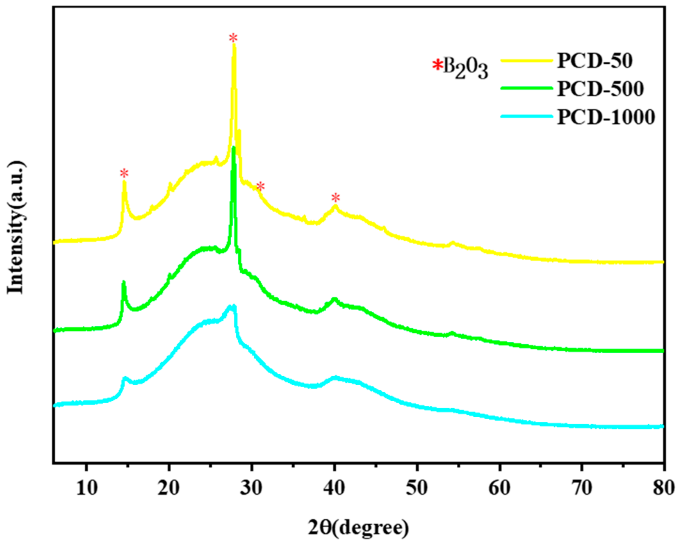

Figure S6 XRD patterns of PCD50, PCD500 and PCD1000, respectively.

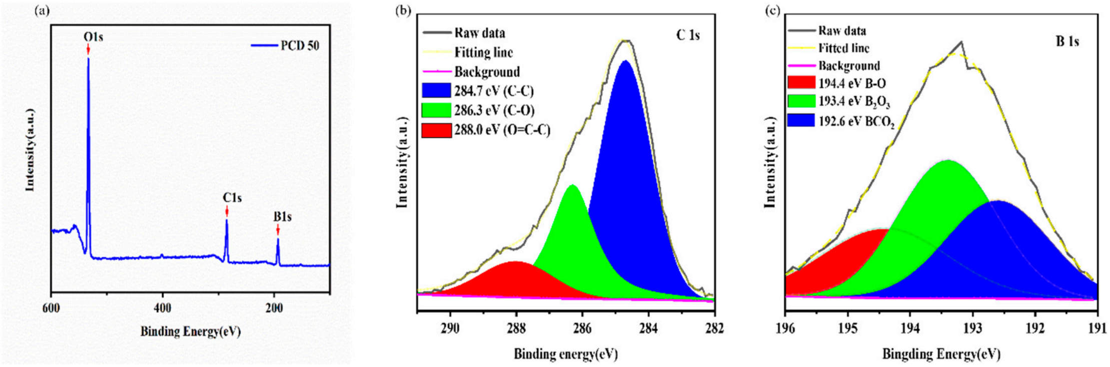

Figure S7 (a) Full scan XPS spectrum of PCD50. HR XPS C 1s (b) and B 1s (c) spectra of PCD50.

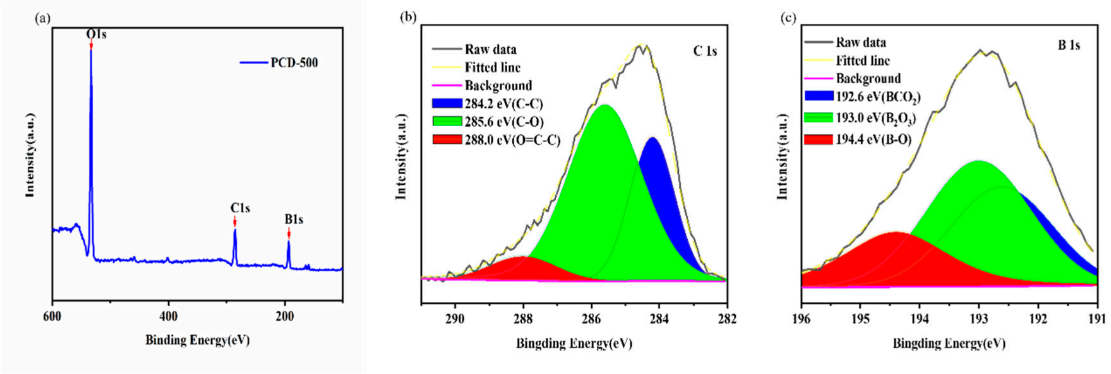

Figure S8 (a) Full scan XPS spectrum of PCD500. HR XPS C 1s (b) and B 1s (c) spectra of PCD500.

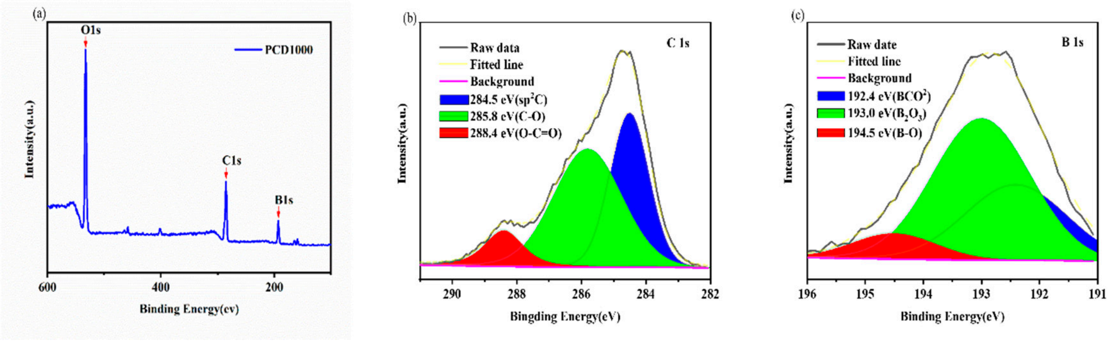

Figure S9 (a) Full scan XPS spectrum of PCD1000. HR XPS C 1s (b) and B 1s (c) spectra of PCD1000.

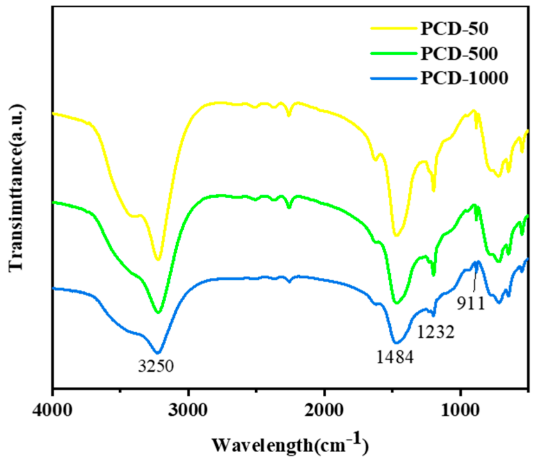

Figure S10 FTIR patterns of PCD50, PCD500 and PCD1000, respectively.

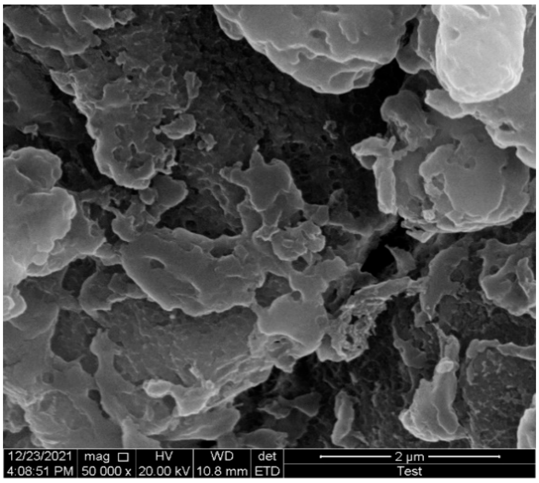

Figure S11 SEM image of PCD100

Table S1. Fluorescence lifetime of PCDs under 365 nm.

PCDs	A1	T1(ns)	A2	T2(ns)	A3	T3(ns)	T_{ave}(ns)
PCD50	1314.064	1.53111	1706.274	4.08068	248.5376	11.19366	5.33
PCD100	3676.45	1.85854	2989.83	4.94927	248.9425	14.6089	5.50
PCD500	5792.112	1.35522	14605.4	3.9698	2578.231	10.16136	5.51
PCD1000	11604.89	1.2001	35590.82	3.88546	5481.684	10.22111	5.41

Table S2. Photophysical lifetime of PCDs under 365 nm.

PCDs	A1	T1(ns)	A2	T2(ns)	A3	T3(ns)	T_{ave}(ns)	T_{ave}(ms)
PCD50	44966.45	8277670	15390.29	75772500	3061.887	518374000	292492397.9	292
PCD100	45058.51	8344550	15339.14	76037900	3049.998	519701000	292762537	293
PCD500	82537.85	10418800	38853.63	62177400	7637.832	383133000	206436458.5	207
PCD1000	54628.6	6565740	19818.77	45523300	3849.948	285742000	151533921.4	152

Table S3. The contents of C, O and B in PCDs determined by XPS.

PCDs	C Content(%)	O Content(%)	B Content(%)
50	22.46	42.75	32.93
100	32.18	38.3	27.45
500	19.55	43.9	34.52
1000	30.33	39.38	27.35

Table S4. The FL emission peaks, RTP emission peaks, and calculated ΔE_{ST} of PCD50 and PCD100 stimulated at 300nm.

	FL peak (nm)	RTP peak (nm)	ΔEST (eV)
PCD50	467	558	0.43
PCD100	470	560	0.42
